# Development and initial validation of the career resilience instrument for CDC emergency responders in China within the context of public health emergencies: based on a survey conducted in Shanghai

**DOI:** 10.3389/fpubh.2024.1327738

**Published:** 2024-03-07

**Authors:** An-Qi Wang, Wen-Di Cheng, Yu-Yan Fu, Ya-Shuang Luo, Juan Li, Hai-Yin Wang, Chun-Lin Jin

**Affiliations:** ^1^School of Public Health, Shandong Second Medical University, Weifang, Shandong, China; ^2^Department of Health Technology Assessment, Shanghai Health Development Research Center (Shanghai Medical Information Center), Shanghai, China; ^3^School of Public Health, Shanghai University of Traditional Chinese Medicine, Shanghai, China

**Keywords:** career resilience, emergency responders, center for disease control and prevention, public health emergency, measurement instrument

## Abstract

**Background:**

China faces various public health emergencies, and emergency responders at the Centers for Disease Control and Prevention (CDC emergency responders) are a mainstay in responding to public health emergencies. Career resilience can help CDC emergency responders to effectively respond to and recover from public health emergencies, but there is no specific measurement instrument available. In this study, we aimed to develop and conduct an initial validation of the career resilience instrument for CDC emergency responders in China within the context of public health emergencies from a process perspective.

**Methods:**

Based on a survey conducted in Shanghai, interpretive phenomenological analysis (IPA), which is a qualitative research approach to describing and analyzing individual experiences, was used to analyze the interview texts to develop the initial career resilience instrument for CDC emergency responders. The initial career resilience instrument was revised through two rounds of expert consultation. Cronbach’s α coefficient and exploratory factor analysis were used to test the reliability and validity of the revised career resilience instrument.

**Results:**

The initial career resilience instrument for CDC emergency responders contained three first-level measurement dimensions, 9 second-level measurement dimensions, and 52 measurement items. After expert consultation, the first-level and second-level measurement dimensions were not revised, 13 measurement items were deleted or revised, and six measurement items were added, resulting in 48 measurement items. The revised career resilience instrument was tested for good reliability and validity.

**Conclusion:**

Career resilience for CDC emergency responders can be regarded as a set of protective factors and dynamic processes that can be cultivated and intervened in cognitive, affective, and behavioral dimensions to improve their ability to respond to and recover from public health emergencies.

## 1 Introduction

In recent years, with the outbreak of severe acute respiratory syndrome (SARS), the global spread of H1N1, the Ebola virus disease (EVD) epidemic in West Africa, the global pandemic of corona virus disease 2019 (COVID-19), and the frequent occurrence of various emergencies, the international community has entered a period of high risk of public health emergencies (1). China, a large country with a population of 1.41 billion, has a high population density and experiences frequent movement of its population within and outside the country, coupled with global warming and rapid urbanization, meaning that China faces greater risks and challenges in terms of public health emergencies (2). Since the outbreak of SARS, China has continued to improve its response mechanism for public health emergencies and accelerate the reform of its public health system (3). The Centers for Disease Control and Prevention (CDC) in China, an important public health agency, have also been in the public spotlight in regard to a series of reforms, mainly concerning managing and responding to public health emergencies through pre-event surveillance and risk assessment, the coordination of resources and provision of epidemiological technical services during events, and post-event summary and analysis to safeguard public health (4, 5). Emergency responders at the Centers for Disease Control and Prevention (CDC emergency responders) are the main force that undertakes the health emergency work of the CDC. CDC emergency responders are those normally undertaking routine work at the CDC, and assuming responsibility for health emergency response and disposal work in the event of public health emergencies, such as comprehensive coordination, sampling, laboratory testing, epidemiological investigation, and disinfection (6). CDC emergency responders face excessive workloads, the risk of viral infection, negative emotions, and turnover intentions during public health emergencies, when individuals need powerful forces to recover to a normal psychological state and work performance level (7, 8). These powerful forces are the same individual characteristics, regulatory processes, and social supports that McLarnon identified in his research, suggesting that they be defined as resilience, where individuals rely on these characteristics, processes, and support systems to recover to pre-event levels of performance and well-being (9).

The application of resilience in the career field is known as “career resilience” and was first proposed by London as a component of career motivation (10). The continued study of career resilience is necessary because every employee experiences adverse events in the workplace, and resilience can help employees cope with and bounce back from adversity to achieve positive outcomes and well-being (11, 12). However, there is not yet a unified perspective on the operational definition of career resilience. The trait perspective views career resilience as a characteristic or ability relating to combatting career adversity, coping with work stress, and adapting to environmental change (13–15). The outcome perspective views career resilience as achieving favorable outcomes in the face of adversity or stress in the workplace, including recovery to a normal work state or growth toward a new ordered state (16, 17). The process perspective views career resilience as the positive and effortful process by which individuals adapt to and recover from adversity in the workplace, involving their initial responses in the early stages of adverse events, individual protective factors, and self-regulatory processes (18, 19). We assume that the process perspective can more comprehensively describe the dynamic development process of resilience, which contains the connotative elements in the trait and outcome perspectives. Therefore, in this study, we attempted to develop the career resilience measurement instrument for CDC emergency responders from a process perspective.

King and Rothstein (20) have provided a more comprehensive and cogent view of career resilience from a process perspective. In the King and Rothstein (20) model, career resilience has been conceptualized as a dynamic process of individual-environment interaction, involving a higher-order, multidimensional construct of cognitive, affective, and behavioral protective factors and self-regulatory processes (20). These resilience processes are provoked by individual initial responses to an adverse event in the workplace and influenced by individual protective factors and social support resources, leading to self-regulation and recovery from an adverse event (21). Specifically, career resilience entails (a) individual protective factors, such as positive cognitive, affective, and behavioral characteristics; (b) social support resources; (c) initial responses following an adverse event; and (d) self-regulatory processes, such as positive self-cognitive, self-affective, and self-behavioral regulation, as well as related recovery outcomes (20). This model was developed to capture the key elements and processes by which individuals recover from adversity in the workplace, suggesting that resilience can be elicited through the management of an individual’s thoughts, feelings, and actions to cope with various adverse events and poor experiences in the workplace (9).

Previous studies rarely explored the connotative elements and measurement instruments of career resilience from a process perspective, much less developed context-and population-specific career resilience instruments with CDC emergency responders as the subject of study. Using the King and Rothstein (20) model as a guide, this study explores where career resilience for CDC emergency responders should be measured in the context of public health emergencies, particularly in the face of major public health emergencies. Thus, the aim of this study was to (a) develop the career resilience instrument for CDC emergency responders, and (b) conduct an initial validation of the developed career resilience instrument.

## 2 Methods

### 2.1 Study design

This study was conducted in three phases: (a) Face-to-face, in-depth, semi-structured interviews were conducted with the sampled CDC emergency responders, and the initial career resilience instrument for CDC emergency responders was generated by analyzing the interview texts. (b) Through consultation with experts in the field of health emergencies, the initial career resilience instrument for CDC emergency responders was revised and improved based on the importance ratings and revision opinions of the experts. (c) The reliability and validity of the revised career resilience instrument for CDC emergency responders were validated.

### 2.2 Sampling and data collection

Participants in this study were included according to the selection criteria provided below, and each participant understood the purpose of this survey and agreed to participate in it. The survey could be terminated at any time if the participant did not want to continue participating in the survey while it was in progress. The participants in this study were from Shanghai, the center city of China, with 16 districts, a total area of 6340.5 square kilometers, and a resident population of 24.76 million in 2022. From March to May 2022, Shanghai was under closed management in response to COVID-19, in which CDC emergency responders played an important role.

First, the criteria for selecting the interview subjects in this study were as follow: we included those who (a) were engaged in health emergency-related work at the CDC for 2 years or more, (b) had participated in health emergency response and disposal of public health emergencies, and worked for a cumulative duration of 14 days or more, and (c) volunteered and agreed to participate in the interviews. In accordance with the above criteria, 10 emergency responders were sampled from each of the three district-level CDCs in central, suburban, and far-suburban Shanghai, serving as the interview subjects for this study, finally, 30 interview subjects were selected. The interview centered on “CDC emergency responders’ literacy characteristics, main tasks, difficulties encountered, ways of overcoming difficulties, changes in thoughts and feelings, adjustments in work status, etc.” Each interview subject was interviewed once in a face-to-face, in-depth, semi-structured interview, with three interviewers using the same interview outline (refer to the detailed interview outline in Additional File 1), which was audio-recorded in its entirety, with the interview subject’s permission. For these three interviewers, one acted as the lead interviewer, and the other two were responsible for transcribing and supplementing. At the end of the interview, it was determined that each interview lasted between 20 and 40 min, and the interview data were organized promptly. If the interview subject did not allow audio-recording, the two interview transcribers compared what was transcribed and, in case of disagreement, could first seek confirmation from the lead interviewer. If the lead interviewer was unable to provide confirmation, the interview subject could be asked to confirm the information again to ensure accuracy and form a complete text. If the interview subject allowed audio-recording, it could be transcribed into a text by listening to the audio-recording repeatedly. Thirty interview texts were eventually collected.

Second, the criteria for selecting the experts to be consulted were as follow: the participants had to have (a) been engaged in health emergency-related work for 5 years or more, (b) carried out research work related to health emergencies, and (c) volunteered and agreed to participate in this survey. Based on the above criteria, 25 experts were sampled for this study, 24 of whom completed the consultation form, and two rounds of consultation were conducted with these experts. The initial career resilience instrument for CDC emergency responders, generated based on the interview text, was subjected to the first round of expert consultation, in which experts scored the importance of each dimension and item on a scale of 1–5, with higher scores representing greater affirmation of significance and importance. The initial career resilience instrument was revised through the first round of expert consultation, and the revised instrument was subjected to the second round of expert consultation, in which, again, the scores were rated on a scale of 1–5 according to importance.

Finally, we compiled the career resilience instrument, as revised through expert consultations, into a questionnaire and validated its reliability and validity after sampling CDC emergency responders to complete the questionnaire. The sampled CDC emergency responders fitted the following criteria: they (a) were involved in responding to public health emergencies, (b) worked in emergency-related operational departments at CDC, and (c) volunteered and agreed to participate in filling out this questionnaire. There are 16 district-level CDCs in Shanghai, and we randomly sampled 80–120 CDC emergency responders using the above criteria from each district CDC to fill out the questionnaire anonymously. For each questionnaire, we performed strict quality control procedures and eliminated questionnaires with incomplete key information and contradictory logic, ultimately leading to 1,286 valid questionnaires.

### 2.3 Data analysis

Interpretive phenomenological analysis (IPA) is a widely used qualitative research approach for describing and analyzing the subjective perceptions and experiences of individuals, revealing the meaning of each individual experience, comparing commonalities and differences, and then reducing the description of the individual experience to the essential elements and features of the things (22). We first used IPA to analyze the collected interview texts. To ensure the reliability of the analysis results, two researchers analyzed the interview texts in a back-to-back format. Where the two researchers’ analyses differed, a third researcher was asked to exercise judgment in order to determine the final analysis results. This study followed the steps of IPA (23, 24): firstly, the interview texts were read repeatedly, and initial notes and comments were made on valuable textual information. Secondly, the repetitive and similar initial notes and comments were merged and summarized to form the main comments, based on which sub-themes were extracted and named. Finally, associations between sub-themes were searched for, similar sub-themes were clustered into theme groups, and these theme groups were streamlined and refined to produce themes. As all the interview texts were analyzed and correlations between themes were explored in the above process, the IPA steps of “analyzing the information of the next subject, looking for thematic patterns between each other” were not repeated.

Second, this study applied the expert positivity coefficient, expert authority coefficient, and expert coordination coefficient to evaluate the expert consultations. The expert positivity coefficient refers to the degree of interest and cooperation of experts in this study, which is reflected through the valid response rate for the expert consultation form, and usually a response rate of 70% or more indicates a good expert positivity coefficient (25). The expert authority coefficient is reflected by the experts’ familiarity with the study and the judgment basis, and the mean value of the familiarity score and judgment basis score is the expert authority coefficient, which is above 0.7, indicating reliable results (25). The familiarity was set at five levels, ranging from “very familiar” to “very unfamiliar,” with scores of 1, 0.8, 0.6, 0.4, and 0.2, respectively, and the judgment basis was divided into four dimensions, namely, work experience, theoretical analysis, reference literature, and intuitive feeling. Each dimension was divided into three levels,namely, large, medium, and small, and the specific scores are shown in Table 1. The expert coordination coefficient is generally calculated using Kendall’s *W* (*W*), which is used to reflect the degree of coordination of the experts’ opinions regarding the content of the consultation. The *W*-value is between 0 and 1, and after several rounds of expert consultation, the *W*-value generally fluctuates at around 0.5. The significance of the *W*-value is tested using *χ*^2^, and a *W*-value that is statistically significant (*p* < 0.05) indicates that the degree of coordination of the opinions among experts is good (25). In addition, we adopted the boundary value method to screen the indicators, and according to the importance ratings (ranging from 1 to 5) and revised opinions of the experts, the indicators with “the mean value of importance less than 4, and the coefficient of variation greater than 0.25″ were deleted or revised (26, 27).

**Table 1:** Scale for assigning scores to the expert judgment basis.

Judgment basis	Scores		
	Large	Medium	Small
Work experience	0.5	0.4	0.3
Theoretical analysis	0.3	0.2	0.1
Reference literature	0.1	0.1	0.1
Intuitive feeling	0.1	0.1	0.1

Third, for the career resilience instrument, as revised by the expert consultations, we used Cronbach’s α coefficient to test its reliability and exploratory factor analysis to determine its structural validity. A Cronbach’s α coefficient greater than 0.7 indicates good reliability, and in exploratory factor analysis, a Kaiser-Meyer-Olkin (KMO) value greater than 0.7 and a *p*-value less than 0.05 indicate that it is suitable for factor analysis (28). In this study, the principal component method was used for factor extraction, the maximum variance method was used for factor rotation, and the number of factors was determined based on the eigenvalue being greater than 1. The items with a factor loading greater than 0.4 were considered appropriate.

## 3 Results

### 3.1 Demographic characteristics

#### 3.1.1 Demographic characteristics of the interview subjects

Of the 30 interviewed subjects, 13 were male, accounting for 43.33%. The majority were 31–35 years old, amounting to 36.67%. The academic qualifications and majors were predominantly a postgraduate degree and preventive medicine, accounting for 63.33 and 93.33%, respectively. The titles and positions were mainly middle level and common staff, accounting for 53.33 and 80.00%, respectively. Working tenure was mostly 6–10 years, accounting for 36.67%. The specific demographic characteristics of the interviewed subjects are shown in Table 2.

**Table 2:** Demographic characteristics of interview subjects (*N* = 30).

Variables	*N* (%)	Variables	*N* (%)
Gender		Title	
Male	13 (43.33)	Vice high level	3 (10.00)
Female	17 (56.67)	Middle level	16 (53.33)
Age (years)		Junior level	8 (26.67)
≤ 30	10 (33.33)	Untitled	3 (10.00)
31–35	11 (36.67)	Position	
36–40	6 (20.00)	Head of the department	6 (20.00)
> 40	3 (10.00)	Common staff	24 (80.00)
Academic qualification		Working tenure (years)	
Postgraduate	19 (63.33)	≤ 5	12 (40.00)
Undergraduate	10 (33.33)	6–10	11 (36.67)
Junior college	1 (3.34)	11–15	5 (16.65)
Major		16–20	1 (3.34)
Preventive medicine	28 (93.33)	> 20	1 (3.34)
Others	2 (6.67)		

#### 3.1.2 Demographic characteristics of the experts consulted

Two rounds of consultation were conducted with 24 experts in this study. The experts consulted were mainly female, accounting for 58.33%, and mostly 36–40 years old, accounting for 41.67%. The academic qualifications and majors were predominantly a postgraduate degree and preventive medicine, accounting for 70.83 and 87.50%, respectively. Titles and positions were mainly middle level and head of the department, accounting for 54.17 and 66.66%, respectively. Working tenure was mostly 11–15 years, accounting for 37.50%. The specific demographic characteristics of the experts consulted are shown in Table 3.

**Table 3:** Demographic characteristics of the experts consulted (*N* = 24).

Variables	*N* (%)	Variables	*N* (%)
Gender		Title	
Male	10 (41.67)	High level	2 (8.33)
Female	14 (58.33)	Vice high level	9 (37.50)
Age (years)		Middle level	13 (54.17)
≤ 35	7 (29.17)	Position	
36–40	10 (41.67)	Head of the institution	1 (4.17)
41–45	4 (16.66)	Head of the department	16 (66.66)
> 45	3 (12.50)	Common staff	7 (29.17)
Academic qualification		Working tenure (years)	
Postgraduate	17 (70.83)	≤ 10	7 (29.17)
Undergraduate	7 (29.17)	11–15	9 (37.50)
Major		16–20	5 (20.83)
Preventive medicine	21 (87.50)	21–25	2 (8.33)
Clinical medicine	1 (4.17)	> 25	1 (4.17)
Health management	2 (8.33)		

#### 3.1.3 Demographic characteristics of the CDC emergency responders sampled from 16 district-level CDCs in Shanghai

Of the 1,286 CDC emergency responders sampled, 543 (42.22%) were male, and there were more participants under the age of 30 and between 31 and 40 years of age, accounting for 38.18 and 37.56%, respectively. The academic qualifications and majors were predominantly undergraduate and preventive medicine, accounting for 65.32 and 79.47%, respectively. The majority of the titles and positions were middle level and common staff, accounting for 41.68 and 85.07%, respectively, while the majority of the working tenure was less than 5 years, accounting for 39.42%. The specific demographic characteristics of the sampled CDC emergency responders are shown in Table 4.

**Table 4:** Demographic characteristics of the CDC emergency responders sampled from 16 district-level CDCs in Shanghai (*N* = 1,286).

Variables	*N* (%)	Variables	*N* (%)
Gender		Title	
Male	543 (42.22)	High level	14 (1.09)
Female	743 (57.78)	Vice high level	119 (9.25)
Age (years)		Middle level	536 (41.68)
≤ 30	491 (38.18)	Junior level	395 (30.72)
31–40	483 (37.56)	Untitled	219 (17.03)
41–50	238 (18.51)	Others	3 (0.23)
> 50	74 (5.75)	Position	
Academic qualification		Head of the institution	5 (0.39)
Postgraduate	368 (28.62)	Head of the department	187 (14.54)
Undergraduate	840 (65.32)	Common staff	1,094 (85.07)
Junior college	50 (3.89)	Working tenure (years)	
High school/ Secondary technical school and below	28 (2.17)	≤ 5	507 (39.42)
Major		6–10	270 (21.00)
Preventive medicine	1,022 (79.47)	11–15	185 (14.39)
Clinical medicine	47 (3.65)	16–20	150 (11.66)
Basic medicine	25 (1.95)	> 20	174 (13.53)
Medical laboratory technology	93 (7.23)		
Health management	32 (2.49)		
Others	67 (5.21)		

### 3.2 Interview text analysis results

IPA was used in this study to analyze the interview texts. The researchers read the interview texts repeatedly to fully familiarize themselves with these texts and acquire an overall impression of the situation described by the interview subjects. On this basis, with an open mind, initial notes and comments were made on the original statements in the interview texts that were related to the theme of this study. The collected interview texts of the 30 interview subjects extracted 567 original statements related to the theme of this study, and these original statements were subjected to initial notes and comments. Subsequently, repetitions and similarities of the 567 initial notes and comments were merged and collated, resulting in 52 main comments. Nine sub-themes were extracted by iterating through and reflecting on the 52 main comments and referring to the King and Rothstein model (20). The nine sub-themes were clustered into three theme groups by searching for correlations between them. The three theme groups were streamlined and refined into three themes with reference to Duchek’s resilience model (29), which elaborates that resilience consists of the ability to anticipate an unexpected event, cope during an unexpected event, and adapt afterward.

#### 3.2.1 Theme 1: anticipatory resilience

In this study, anticipatory resilience referred to the CDC emergency responders’ resilience characteristics possessed before public health emergencies, which could be a guide and facilitator allowing individuals to clearly define their roles, maintain stable emotions, and quickly enter the “wartime” state when emergencies occurred. Anticipatory resilience included four sub-themes: career cognitive characteristics, career affective characteristics, career behavioral characteristics, and resource acquisition ability.

##### 3.2.1.1 Sub-theme 1: career cognitive characteristics

The career cognitive characteristics of CDC emergency responders mainly concerned self-efficacy, sense of meaning in work, etc., which could play a guiding and normative role with respect to individuals changing their cognition of roles, clarifying their role positions, and taking on their role’s function in response to public health emergencies. The career cognitive characteristics contained seven main comments, which are presented in Supplementary Table S1 in Additional File 1.

##### 3.2.1.2 Sub-theme 2: career affective characteristics

The CDC emergency responders were characterized by stable emotions, calmness, and patience with respect to dealing with problems and a passion for work. These characteristics could serve as facilitators and safeguards with regard to allowing individuals to quickly regulate negative emotions and focus on tasks during a response to public health emergencies. There were four main comments on the career affective characteristics, which are shown in Supplementary Table S1 in Additional File 1.

##### 3.2.1.3 Sub-theme 3: career behavioral characteristics

The career behavioral characteristics of CDC emergency responders primarily involved planning, execution, adaptability, innovation, and learning, which could play leading and protective roles in enabling individuals to quickly adjust to the behaviors required in a “wartime” scenario when responding to public health emergencies. The career behavioral characteristics included nine main comments, which are shown in Supplementary Table S1 in Additional File 1.

##### 3.2.1.4 Sub-theme 4: resource acquisition ability

The resource acquisition ability of CDC emergency responders included both instrumental and affective resource acquisition abilities, with six main comments on resource acquisition ability, as shown in Supplementary Table S1 in Additional File 1.

#### 3.2.2 Theme 2: coping resilience

In this study, coping resilience referred to the active self-regulation of CDC emergency responders during their response to public health emergencies, adapting to the emergency work state, and completing emergency tasks flexibly and efficiently. Coping resilience involved four sub-themes: stress responses, self-cognitive regulation, self-affective regulation, and self-behavioral regulation.

##### 3.2.2.1 Sub-theme 5: stress responses

In the initial occurrence of a public health emergency, the CDC emergency responders faced stress with a range of responses, including behavioral, cognitive, emotional, and physiological responses. The stress responses consisted of five main comments, as shown in Supplementary Table S2 in Additional File 1.

##### 3.2.2.2 Sub-theme 6: self-cognitive regulation

Self-cognitive regulation involved CDC emergency responders’ efforts with respect to re-examining a problem, adjusting their cognition about unfavorable circumstances, and changing their ways of conceiving the problems while responding to public health emergencies in order to adjust and adapt to emergencies promptly. There were four main comments on self-cognitive regulation, as shown in Supplementary Table S2 in Additional File 1.

##### 3.2.2.3 Sub-theme 7: self-affective regulation

During the response phase of public health emergencies, CDC emergency responders regulated their negative emotions and work stress and maintained a calm, stable, optimistic, and confident emotional state. There were five main comments on self-affective regulation, which can be seen in Supplementary Table S2 in Additional File 1.

##### 3.2.2.4 Sub-theme 8: self-behavioral regulation

During the response phase of public health emergencies, the CDC emergency responders quickly adjusted from a routine behavioral status to “wartime” emergency behavior to complete the emergency tasks. There were eight main comments on self-behavioral regulation, which can be seen in Supplementary Table S2 in Additional File 1.

#### 3.2.3 Theme 3: recovery resilience

In this study, recovery resilience referred to the ability the of CDC emergency responders to recover from the “wartime” state after the public health emergency response had ceased, as well as their ability to summarize and learn from the emergencies and reflect on their improvement. Recovery resilience included one sub-theme: adaptive outcomes.

##### 3.2.3.1 Sub-theme 9: adaptive outcomes

Adaptive outcomes referred to the ability of CDC emergency responders to recover to their original state, learn and reflect on emergencies, and even reach a new state of equilibrium after a public health emergency response was completed. There were four main comments on the adaptive outcome, as shown in Supplementary Table S3 in Additional File 1.

#### 3.2.4 Initial career resilience instrument

The CDC emergency responders’ connotative components of career resilience in the context of public health emergencies were collected based on the interview text and included the career characteristics of CDC emergency responders, individual stress responses and state regulation during public health emergencies, and recovery and growth after public health emergencies. Therefore, this study developed the initial career resilience instrument for CDC emergency responders by collating and analyzing the interview texts, with 52 main comments serving as the specific measurement items, nine sub-themes serving as the second-level measurement dimensions, and three themes serving as the first-level measurement dimensions.

### 3.3 Expert consultation results

We compiled the initial career resilience instrument for CDC emergency responders into an expert consultation form, where experts scored the importance of each measurement dimension and each measurement item on a scale of 1–5, with higher scores being considered more appropriate for measuring career resilience.

#### 3.3.1 Basic information on expert consultations

Twenty-five expert consultation forms were sent out for the first round of expert consultation, of which 24 were returned, representing a valid response rate of 96%. In the second round of expert consultations, 24 expert consultation forms were sent out and 24 were returned, with a valid response rate of 100%. The valid response rates for both rounds of expert consultation were above 70%, indicating a high expert positivity coefficient. The authority coefficients of the two rounds of expert consultation were 0.880 and 0.886, respectively, which were both above 0.7, indicating that the results of expert consultation were reliable. The expert coordination coefficients for the first-level and second-level measurement dimensions were 0.420 and 0.423 in the first round of expert consultation and did not change in the second round. The expert coordination coefficients of the measurement items were 0.306 and 0.421 in the first and second rounds of expert consultation, respectively. In the second round of expert consultation, the expert coordination coefficients of each measurement dimension and measurement item were close to 0.5, with *p* < 0.001, indicating that the experts’ consultation opinions were relatively consistent. Table 5 presents basic information on the expert consultations.

**Table 5:** Basic information on expert consultation.

	The first round of expert consultation	The second round of expert consultation
Expert positivity coefficient	96%	100%
Expert authority coefficient	0.880	0.886
Familiarity score	0.842	0.850
Judgment basis score	0.917	0.921
Expert coordination coefficient		
The first-level measurement dimensions	0.420^***^	0.420^***^
The second-level measurement dimensions	0.423^***^	0.423^***^
The measurement items	0.306^***^	0.421^***^

*** *p* < 0.001.

#### 3.3.2 Expert revisions to the initial instrument

Measurement dimensions and items were deleted or revised based on the following criterion: a “mean value of importance less than 4 and coefficient of variation greater than 0.25.” The results of both rounds of expert consultation showed that the mean values of importance for the first-level and second-level measurement dimensions were greater than 4 and that the coefficients of variation were less than 0.25, so they were not deleted or revised.

For the measurement items, in the first round of expert consultation, there were 13 items with a mean value of importance less than 4 and a coefficient of variation greater than 0.25. These 13 items were deleted or revised. For example, the item “Ensure coordination of tasks and resources in health emergency response” had a mean value of 2.958 and a coefficient of variation of 0.323, and the experts recommended its deletion because the majority of emergency responders’ tasks and resources are carried out according to regulations or are assigned by their leaders, in which individual coordination plays a minor role, and thus they item does not fully capture the meaning of career resilience. The experts’ specific revision opinions on these items are presented in Supplementary Table S4 in Additional File 1.

Moreover, in the first round of expert consultation, the experts suggested adding the following six items. For the dimension “Career behavioral characteristics,” one item for “Adapting proactively to changes in health emergency work” was added. For the dimension “Self-cognitive regulation,” two items for “During the response to public health emergencies, controlling negative thoughts and believing in good results” and “During the response to public health emergencies, adjusting thoughts and focusing on problems that need to be solved” were added. For the dimension “Self-behavioral regulation,” three items for “During the response to public health emergencies, being able to adjust quickly from the usual work pace to high-intensity work,” “During the response to public health emergencies, proactively reconciling work and family and focusing on the completion of emergency tasks,” and “During the response to public health emergencies, adjusting the way of obtaining information, and proactively collecting and analyzing information on emergencies” were added.

After revision through the first round of expert consultation, all measurement items were subjected to a second round of expert consultation, with a mean value of importance greater than 4 and a coefficient of variation less than 0.25 for all of them, so none of the items were deleted, and only minor revisions were made to the wording and presentation of a few items. The career resilience instrument for CDC emergency responders, as revised by the expert consultation, contained three first-level measurement dimensions, 9 second-level measurement dimensions, and 48 measurement items.

### 3.4 Reliability and validity test results

#### 3.4.1 Reliability test results

The reliability of the career resilience instrument for CDC emergency responders, as revised via expert consultations, was tested using Cronbach’s α coefficient. The results showed that the Cronbach’s α coefficient for the entire instrument was 0.969; the Cronbach’s α coefficient for the three first-level dimensions of anticipatory resilience, coping resilience, and recovery resilience were 0.956, 0.921, and 0.910 respectively; and the Cronbach’s α coefficient for the 9 second-level dimensions of career cognitive characteristics, career affective characteristics, career behavioral characteristics, resource acquisition ability, stress responses, self-cognitive regulation, self-affective regulation, self-behavioral regulation, and adaptive outcomes were 0.928, 0.913, 0.954, 0.925, 0.871, 0.943, 0.956, 0.961, and 0.910, respectively. The Cronbach’s α coefficients for each dimension were all above 0.8, indicating good reliability of the instrument. Table 6 demonstrates the Cronbach’s α coefficients for each dimension.

**Table 6:** The Cronbach’s α coefficients for each dimension.

The first-level dimension	Number of items	Cronbach’s α	The second-level dimension	Number of items	Cronbach’s α
Anticipatory resilience	24	0.956	Career cognitive characteristics	7	0.928
			Career affective characteristics	4	0.913
			Career behavioral characteristics	8	0.954
			Resource acquisition ability	5	0.925
Coping resilience	20	0.921	Stress responses	3	0.871
			Self-cognitive regulation	4	0.943
			Self-affective regulation	5	0.956
			Self-behavioral regulation	8	0.961
Recovery resilience	4	0.910	Adaptive outcomes	4	0.910

#### 3.4.2 Validity test results

The exploratory factor analysis was first conducted on the instrument of anticipatory resilience, and the results showed that the KMO value was 0.941, with a *p*-value of less than 0.001, rendering it suitable for factor analysis. Four factors were extracted from the 24 items, which were in line with the pre-defined results, and named career behavioral characteristics (with eight items), career cognitive characteristics (with seven items), resource acquisition ability (with five items), and career affective characteristics (with four items). The variance percentages of the four factors were 24.522, 21.171, 16.952, and 12.942%, respectively, and the factor loading values of the items corresponding to the four factors were 0.678 ~ 0.850, 0.764 ~ 0.858, 0.697 ~ 0.803, and 0.701 ~ 0.806, respectively. All the factor loading values were above 0.65, indicating good structural validity. Table 7 presents the factor loading values of anticipatory resilience items.

**Table 7:** Factor loading matrix of anticipatory resilience items.

Item	Career behavioral characteristics	Career cognitive characteristics	Resource acquisition ability	Career affective characteristics	Percentage of variance (%)
Career behavioral characteristics item 2	0.850	0.120	0.183	0.188	24.522
Career behavioral characteristics item 6	0.790	0.109	0.230	0.220	
Career behavioral characteristics item 7	0.776	0.184	0.314	0.178	
Career behavioral characteristics item 4	0.775	0.169	0.321	0.258	
Career behavioral characteristics item 8	0.748	0.161	0.325	0.211	
Career behavioral characteristics item 1	0.737	0.203	0.256	0.308	
Career behavioral characteristics item 3	0.721	0.191	0.416	0.261	
Career behavioral characteristics item 5	0.678	0.197	0.445	0.257	
Career cognitive characteristics item 4	0.103	0.858	0.042	0.115	21.171
Career cognitive characteristics item 2	0.083	0.855	0.077	0.154	
Career cognitive characteristics item 6	0.106	0.820	0.203	0.107	
Career cognitive characteristics item 3	0.105	0.814	0.059	0.084	
Career cognitive characteristics item 1	0.145	0.811	−0.010	0.074	
Career cognitive characteristics item 5	0.203	0.772	0.138	0.064	
Career cognitive characteristics item 7	0.153	0.764	0.194	0.098	
Resource acquisition ability item 4	0.377	0.107	0.803	0.230	16.952
Resource acquisition ability item 2	0.401	0.134	0.775	0.206	
Resource acquisition ability item 5	0.422	0.143	0.751	0.231	
Resource acquisition ability item 1	0.345	0.101	0.708	0.236	
Resource acquisition ability item 3	0.276	0.175	0.697	0.279	
Career affective characteristics item 3	0.254	0.118	0.321	0.806	12.942
Career affective characteristics item 2	0.368	0.176	0.273	0.765	
Career affective characteristics item 1	0.346	0.192	0.186	0.753	
Career affective characteristics item 4	0.363	0.194	0.345	0.701	

Then, an exploratory factor analysis was conducted on the instrument of coping resilience, which needed to be normalized as the three items of the pre-set stress responses were set in reverse, and the results after processing showed that the KMO value was 0.907, with a *p*-value of less than 0.001, rendering it suitable for factor analysis. Four factors were extracted from the 20 items, which were in line with the pre-defined results, and named self-behavioral regulation (with eight items), self-affective regulation (with five items), self-cognitive regulation (with four items), and stress responses (with three items). The variance percentages of the four factors were 31.413, 21.567, 17.208, and 11.976%, respectively, and the factor loading values of the items corresponding to the four factors were 0.819 ~ 0.877, 0.795 ~ 0.902, 0.858 ~ 0.875, and 0.821 ~ 0.895,respectively.All the factor loading values were above 0.75, indicating good structural validity. Table 8 presents the factor loading values of coping resilience items.

**Table 8:** Factor loading matrix of coping resilience items.

Item	Self-behavioral regulation	Self-affective regulation	Self-cognitive regulation	Stress responses	Percentage of variance (%)
Self-behavioral regulation item 2	0.877	0.177	0.148	0.133	31.413
Self-behavioral regulation item 5	0.875	0.156	0.177	0.157	
Self-behavioral regulation item 6	0.853	0.172	0.146	0.159	
Self-behavioral regulation item 7	0.848	0.172	0.089	0.125	
Self-behavioral regulation item 3	0.838	0.160	0.153	0.103	
Self-behavioral regulation item 1	0.837	0.200	0.184	0.120	
Self-behavioral regulation item 8	0.834	0.163	0.139	0.153	
Self-behavioral regulation item 4	0.819	0.170	0.176	0.158	
Self-affective regulation item 3	0.179	0.902	0.204	−0.077	21.567
Self-affective regulation item 4	0.218	0.894	0.207	−0.027	
Self-affective regulation item 2	0.207	0.888	0.214	−0.046	
Self-affective regulation item 5	0.198	0.884	0.179	−0.071	
Self-affective regulation item 1	0.232	0.795	0.237	−0.026	
Self-cognitive regulation item 2	0.196	0.237	0.875	−0.089	17.208
Self-cognitive regulation item 3	0.225	0.254	0.868	−0.056	
Self-cognitive regulation item 1	0.195	0.189	0.867	−0.040	
Self-cognitive regulation item 4	0.198	0.272	0.858	−0.087	
Stress responses item 2	0.256	−0.038	−0.053	0.895	11.976
Stress responses item 3	0.222	0.001	−0.031	0.854	
Stress responses item 1	0.188	−0.176	−0.152	0.821	

Finally, an exploratory factor analysis was conducted on the instrument of recovery resilience, and the results showed that the KMO value was 0.836, with a *p*-value of less than 0.001, rendering it suitable for factor analysis. Since only one factor was extracted, no further factor rotation was performed, which was in line with the pre-defined results, and named adaptive outcomes (with four items). The cumulative variance percentage of this single factor was 79.115%, and the factor loading values of each item ranged from 0.865 to 0.911. All the factor loading values were above 0.85, indicating good structural validity. Table 9 presents the factor loading values of recovery resilience items.

**Table 9:** Factor loading matrix of recovery resilience items.

Item	Adaptive outcomes	Percentage of variance (%)
Adaptive outcomes item 2	0.911	79.115
Adaptive outcomes item 3	0.908	
Adaptive outcomes item 1	0.873	
Adaptive outcomes item 4	0.865	

## 4 Discussion

### 4.1 Summary of main findings

Career resilience is a means of helping CDC emergency responders to recover to normal functioning and performance after experiencing public health emergencies, especially major public health emergencies. A growing number of studies are pointing to the widespread impact of developing a validated career resilience assessment instrument, which can guide the design of resilience intervention strategies to fully exploit the effective functioning and optimal performance of resilience when experiencing an adverse event in the workplace (9, 30). Using the King and Rothstein (20) model as a theoretical guide, this study is the first to develop a career resilience instrument for CDC emergency responders in China within the context of public health emergencies from a process perspective and to conduct an initial validation of the developed instrument.

We first sampled 30 CDC emergency responders for face-to-face, in-depth, semi-structured interviews in three district-level CDCs in Shanghai. The interview texts were collated and analyzed using IPA, and 52 main comments, nine sub-themes, and three themes were eventually extracted, from which we constructed the initial career resilience instrument for CDC emergency responders. Secondly, we compiled the initial career resilience instrument into an expert consultation form and invited 24 experts to conduct two rounds of consultation. The expert positivity coefficients, expert authority coefficients, and expert coordination coefficients were all within the ideal value range, and revisions were proposed for 13 measurement items, while six measurement items were added. Further, we compiled the instrument revised by experts into a questionnaire and randomly sampled 1,286 CDC emergency responders in Shanghai, using the questionnaires they filled in for reliability and validity analyses. Additional, reliability and structural validity were examined using Cronbach’s α coefficient and exploratory factor analysis, respectively, which are widely used in the development and validation of instruments (9, 28). The results of this study showed that all the Cronbach’s α coefficients were above 0.85, and the factor loadings in exploratory factor analysis were above 0.65, and there were no double loadings, indicating good results for reliability and validity validation. In this study, the career resilience instrument for CDC emergency responders with three first-level measurement dimensions, 9 second-level measurement dimensions, and 48 measurement items was generated.

### 4.2 Theoretical implications

The theoretical implication of this study is guided by the King and Rothstein (20) model to develop the career resilience instrument for CDC emergency responders in China within the context of public health emergencies from a process perspective. Most previous studies developed instruments to measure career resilience from the trait and outcome perspectives. The trait perspective is a static and isolated way to assess career resilience, which tends to ignore the environmental or contextual factors of individuals’ recovery from adversity, and does not adequately consider the dynamic and developmental attributes of resilience (31, 32). The outcome perspective is a simple way to assess career resilience, and the outcomes achieved in different situational contexts and adverse events are also quite variable (31, 32). Therefore, considering the limitations of the trait and outcome perspectives, in this study, we developed the career resilience measurement instrument from a process perspective, describing protective individual characteristics, internal and external interactions and self-regulatory processes, and adaptive outcomes and employing a variable-centered approach to reveal the dynamic process of an individual’s breakdown from equilibrium to reintegration into equilibrium. In addition, career resilience in the healthcare field has mostly been studied with nurses, and primary healthcare professionals (28, 33–37), while basically, no studies are available regarding CDC emergency responders. This study introduces career resilience to the group of CDC emergency responders in hopes of focusing on the mental health and work status of this group, so that they can better respond to public health emergencies and protect public health.

### 4.3 Practical implications

Resilience is not an entirely innate and unchanging characteristic or trait, on the contrary, it stems from the normative functioning of the human adaptive system and is constituted by ordinary rather than specialized processes, meaning that it can be cultivated (38). This provides the possibility of intervening and cultivating career resilience and a more positive prospect for employees to adapt to various complex work situations. The following provides some practical implications around the exploration of this study.

Firstly, in terms of cognition, it is important to improve self-efficacy through the continuous upgrading of professional skills and actively perceive the value and meaning of work to enrich job satisfaction (39). These actions are conducive to the fact that CDC emergency responders can perceive the good side of an undesirable work environment, refrain from thinking nonsense, and make cognitive regulations in the event of an adverse event such as a public health emergency. Secondly, in terms of affection, health professionals involved in the response to emerging infectious disease outbreaks and major public health emergencies may often feel nervous, fearful, anxious, and powerless (40). This suggests that CDC emergency responders need to learn how to control their emotions and find ways to release their negative emotions, such as confiding in others and being physically active, which can help to regulate negative emotions during emergencies. It has been confirmed that emotional regulation is an unavoidable form of self-regulation when individuals are challenged, and it is used in combination with self-cognitive and self-behavioral regulations to maintain an internal equilibrium (41). Thirdly, in terms of behavior, CDC emergency responders need to acquire cutting-edge knowledge, constantly try new ways to solve problems, prioritize, and plan and schedule work well. One study noted that a sense of control over work schedules is a strong predictor of emotional resilience (42). These can help CDC emergency responders to make timely behavioral adjustments and carry out the actions required for emergencies.

Fourthly, it has been noted that good social networks, such as family support, affirmation from colleagues and leaders, and teamwork, are strongly associated with high levels of resilience (43, 44). This indicates that individuals should actively build a good social network and maintain a balance between interpersonal and work environments. Fifthly, CDC emergency responders should also actively learn to take stock of their work experience after an emergency response is completed. This can be achieved by reconceptualizing work and life, changing priorities, improving interpersonal relationships, enhancing professional competence, and learning ways in which to regulate and release emotions in order to increase resilience levels and respond to more challenges in the future (45).

## 5 Limitations and future research

Although this study has strengths, it still has some limitations. Firstly, the respondents for this study were from Shanghai and nationwide was not conducted, which somewhat interferes with the reliability and generalizability of this study. Therefore, there is a need to sample more areas of China in the future to further test the reliability and validity of this instrument, thereby accumulating more evidence regarding the validity and use of the developed career resilience instrument and determining its reliability and validity for a wider sample. In addition, the use this instrument to assess career resilience among CDC emergency responders in other countries will be attempted to expand its use if possible. Secondly, in this study, we only developed a career resilience instrument for CDC emergency responders and did not explore the antecedent and outcome variables of career resilience in depth. Therefore, future research is required to further explore the influencing factors and outcome effects of CDC emergency responders’ career resilience, which will also serve to further refine this career resilience instrument.

## 6 Conclusion

In this study, we attempted to develop the career resilience instrument for CDC emergency responders and initially validated its good reliability and validity. This study further opens up the application field of career resilience, and provides additional ways and support for assessing career resilience. This study also shows that career resilience can be regarded as a set of protective factors and dynamic processes that can be acquired through the cultivation of individual characteristics and self-regulation in cognitive, affective, and behavioral dimensions, enabling effective responses to and recovery from adverse events in the workplace.

## Data availability statement

The original data presented in this study has been included in the article, further inquiries can be directed to the corresponding authors on reasonable request.

## Author contributions

A-QW: Conceptualization, Data curation, Formal analysis, Investigation, Methodology, Project administration, Software, Validation, Writing – original draft, Writing – review & editing. W-DC: Conceptualization, Data curation, Formal analysis, Investigation, Methodology, Software, Writing – original draft. Y-YF: Conceptualization, Data curation, Formal analysis, Investigation, Methodology, Project administration, Software, Writing – original draft. Y-SL: Conceptualization, Data curation, Formal analysis, Investigation, Methodology, Software, Writing – original draft. JL: Conceptualization, Data curation, Formal analysis, Investigation, Methodology, Software, Writing – original draft. H-YW: Conceptualization, Funding acquisition, Project administration, Resources, Supervision, Validation, Visualization, Writing – review & editing. C-LJ: Conceptualization, Funding acquisition, Project administration, Resources, Supervision, Validation, Visualization, Writing – review & editing.
