# Supplementary material for: Development and initial validation of the career resilience instrument for CDC emergency responders in China within the context of public health emergencies: based on a survey conducted in Shanghai
Source: Front Public Health. 2024 Mar 7;12:1327738. doi: 10.3389/fpubh.2024.1327738 (PMC10955067; doi:10.3389/fpubh.2024.1327738)
Supplement: Supplementary file 1 [file Data_Sheet_1.docx]

**interview outline**

**1、What qualities or characteristics do you think a CDC emergency responder should have? You can talk about them from multiple perspectives, such as cognitive, affective, and behavioral. What role do these qualities or characteristics play in emergency response? What factors affect them?**

**2、What preventive preparations have you made before the occurrence of public health emergencies (e.g., participating in emergency exercises, familiarizing yourself with the emergency plan, monitoring and collecting information, etc.)? What is your attitude towards these tasks (e.g., proactive, careful)? What factors affect your attitude or behavior?**

**3、What tasks have you undertaken in the emergency response to public health emergencies (e.g. epidemiological investigations, on-site control of emergencies, emergency inoculation, etc.)? What are the differences between these tasks and your usual work duties? How did you adjust from your usual work situation to your emergency response role? What factors affect your role adjustment or emergency response performance?**

**4、What difficulties/stresses have you encountered during the emergency response to public health emergencies (e.g., lack of professional competence, high work pressure, risk of infection, etc.)? How do these difficulties/pressures affect your mood or working condition? How have your psychological feelings and cognitive thoughts changed during this process? How did you make positive adjustments?**

**5、What are the biggest gains you have made in the emergency response to public health emergencies? How will these gains affect your daily work or future work?**

**6、After the emergency response to a public health emergency, what summary work have you done (e.g. self-evaluation, work report, paper writing, etc.)? How did this work affect your work status afterward?**

**Table S1 Main comments and representative quotes from the anticipatory resilience**

| Sub-theme | Main Comments | Representative quotes |
| --- | --- | --- |
| Career cognitive characteristics | Believing that the knowledge and skills acquired by oneself are able to perform the health emergency work | *“I am very confident in my professionalism, and I basically have mastered the knowledge and skills required for health emergency work, but of course, I still have to continue to learn and keep up with the times.”* |
|  | Believing that one can overcome the difficulties and pressures of health emergency work. | *“The stress is all there. I like sports and usually release stress with a game of basketball. I don’t dare to say anything else, but I’m still confident that I can do a good job of reducing my stress.”* |
|  | Being able to detect and judge public health risk signals | *“Emergency responders can detect anomalies or identify certain signals through information monitoring, and the leadership praised me for my keen insight and judgment in information.”* |
|  | Believing that one can react quickly and flexibly in emergencies | *“According to their words, I’m a fairly nimble person who can sometimes make quick shifts. I think I’m not bad at all, I’m flexible in dealing with emergencies.”* |
|  | Being positive significance for individuals in health emergency work | *“For me personally, learning how to change my mindset during work is more significant than learning those specific matters.”* |
|  | Helping individuals to better recognize themselves in health emergency work | *“Health emergency work can be a little bit to explore my potential and a deeper understanding of myself. For example, this time, the wearing of protective clothing and goggles caused pressure damage to my facial skin, but even so, I worked continuously. I wore the protective suit for ten hours. I thought I couldn't stand it before, but I carried it off.”* |
|  | Believing that engaging in health emergency work can reflect one’s social contribution and value | *“I feel that I have made a great contribution. The press and media coverage, and the people’s appreciation, made me realize the value of health emergency work, which is hard work but more of a privilege.”* |
| Career affective characteristics | Keeping calm even when things go wrong in the health emergency work | *“The actual health emergency work is more difficult than what is present in the exercises and training, one has to stay calm in any situation to deal with the problem. For example, when the supporting team arrived and the work was not going well because they were not familiar with the situation, we should not be anxious and annoyed, but should solve the problem.”* |
|  | Keeping a normal mind in the face of uncertainties in health emergency work | *“Whether it would get infected and when it would be unblocked, we didn’t know all these. Many factors are uncertain, we still need to put a good mind, with a normal mind to face.”* |
|  | Not having large fluctuations in emotions due to changes in health emergency work conditions | *“No matter how bad the working environment conditions are, you can’t let your emotions take you to work. For example, I was supporting nucleic acid testing in a high-risk area, the working environment was very unfamiliar, and food and accommodation in the workplace without the comfort of home, more or less fluctuations in the mind, but the good thing is that my psychological quality is considered to be good, the mood did not have too much impact, and immediately threw myself into the work.”* |
|  | Working with enthusiasm even when there are various risks and challenges in health emergency work | *“If there is a war, the call will come, the war will be won, which is also our faith in the work, but also a passion.”* |
| Career behavioral characteristics | Being able to set goals for oneself in health emergency work according to the requirements of the position duties and responsibilities | *“To set goals for yourself, do things with planning, every step to do what is clear in mind.”* |
|  | Ensuring that tasks and resources are coordinated in the health emergency work | *“Don’t just focus on your tasks, but also learn how to coordinate your work.”* |
|  | Accomplishing health emergency tasks in accordance with the order of priority | *“As a person who has been working for so many years, which things are light and which things are heavy, this point can still be pinched clearly, and then do things after the distinction.”* |
|  | Being able to take the initiative to participate in all kinds of health emergency work | *“I voluntarily signed up to participate in the 24-hour mobile phone were switched on, ready to standby.”* |
|  | Taking risks and completing tasks with uncertain results in health emergency work | *“There are many risks in health emergency work, and the biggest one is the risk of infection. COVID-19 in the beginning, the pathogenesis and transmission route were not very clear, we could be infected at any time, even so, we have to take the risk, and put down all the distractions to work seriously.”* |
|  | Changing ways of thinking to solve the problems in health emergency work | *“I think it is very important for us to have the courage to argue and question, to try to break the previous rules and regulations, and to use new ways of thinking to solve problems.”* |
|  | Taking the initiative to learn theoretical knowledge and practical skills related to health emergency work | *“At that time, I felt that the knowledge of health emergencies was still not enough when I used it, and I should strengthen my learning. In fact, those operations are only the basic content, but also need to learn how to carry out emergency management.”* |
|  | Participating actively in health emergency exercises and training | *“This also points to a very important point, that we practice less during the usual state. We need to be prepared for all kinds of scenarios.”* |
|  | Learning and training from peers on problems in health emergency work | *“We need to be good at using resources to learn and ask questions if we don’t understand. There’s nothing to be ashamed of when asking for advice from someone with experience.”* |
| Resource acquisition ability | Being able to utilize one’s ability to obtain the required emergency supply to support the individual in completing health emergency work | *“After COVID-19 happened, most of the factories were shut down, and the protective supplies were insufficient. The head of our department got us a batch of supplies through his connections, and we were very happy.”* |
|  | Being able to make use of one’s interpersonal network to obtain professional assistance when needed for health emergency work | *“The last time I did those sample tests, I wasn’t able to do it by myself, so I asked my leader for instructions, but luckily someone helped me along”* |
|  | Being able to obtain technical support and operational guidance when encountering difficulties in health emergency work through active personal communication and endeavor | *“I'm very lucky to have met a good teacher who has been teaching me since I came here.”* |
|  | Being able to gain understanding from family to alleviate negative feelings in health emergency work | *“There are old people and children at home who need to be taken care of, so it’s not true to say that I’m not scared. One day I gave my son a call, and he said Mummy you are so brave, I seem to feel better in my heart, more at ease.”* |
|  | Being able to get help from friends to alleviate negative feelings in health emergency work. | *“Chatting with friends, many of whom are peers and know the experience well, and receiving some encouragement and support, the psychological burden was much lighter”* |
|  | Being able to obtain recognition and affirmation from colleagues/leaders through serious work. | *“These changes and efforts were also seen by the leadership, and after being praised by the leadership, I was more proactive in my work.”* |

**Table S2 Main comments and representative quotes from the coping resilience**

| Sub-theme | Main Comments | Representative quotes |
| --- | --- | --- |
| Stress responses | Being able to report and analyze events information as required when public health emergencies have just occurred | *“In one work, I saw a clear upward trend in the data, and even saw a peak, I made an immediate decision to report with the leader.”* |
|  | Being able to quickly and accurately carry out health emergency tasks when public health emergencies have just occurred | *“The response plan was defined, and following the assignment of tasks, it was possible to immediately go into epidemiological investigations, sampling, etc.”* |
|  | Perceiving as terrible when public health emergencies have just occurred | *“We all compared this epidemic with SARS at the beginning, but it was worse than that, and it was really bad.”* |
|  | Feeling unusually anxious and scared when public health emergencies have just occurred | *“At the beginning, I was a little bit nervous, scared and anxious.”* |
|  | Having trouble eating or sleeping when public health emergencies have just occurred | *“At the beginning, I wore protective clothing and goggles, and I worked for ten hours at a time, and I was very tired when I came back from work. But couldn’t eat, couldn’t sleep, woke up after a short sleep.”* |
| Self-cognitive regulation | During the response to public health emergencies, being more confident in completing the emergency tasks assigned by superiors | *“The subjects of the epidemiological survey came from different countries and the language barrier was a big problem. I was confident in myself, I spent more time and patience. I completed all the tasks required by my superiors on time and following the standard.”* |
|  | During the response to public health emergencies, seeing the positive side of an unfavorable situation | *“I wondered why I had to go to high-risk areas every time, but then I thought it would be good for God to send me there.”* |
|  | During the response to public health emergencies, seeing difficult tasks as the accumulation of experience, and making every effort to complete emergency tasks | *“This was a battle without smoke, all the difficulties were to promote my growth.”* |
|  | During the response to public health emergencies, seeing the stress as an investment in value | *“Through this emergency mission, I have strengthened my ability to resist* *stress and learned to transform and release* *stress, which is a training and growth for me.”* |
| Self-affective regulation | During the response to public health emergencies, being able to regulate the nervousness and anxiety encountered and remain calm | *“There was a brief period of discomfort and anxiety at the beginning. As I became more and more familiar with the workflow, I adjusted quite well and dealt with the problem calmly and comfortably.”* |
|  | During the response to public health emergencies, being able to regulate the fear and dread encountered and remain sensible | *“We took pharyngeal swabs in a high-risk area, and after suffering sputum splashes from the isolators, we were afraid. but the tasks that followed were one after another, so we had to adjust this fear immediately, and thought about the fact that we were still safe in our protective clothing. We needed to look at this matter scientifically and rationally”* |
|  | During the response to public health emergencies, being able to regulate the loneliness and helplessness encountered and maintain optimism | *“I didn’t go home when I was off, and it was like being cut off from the outside world, so it did feel like a sense of loneliness. But it’s okay, I was busy and tired at work, so I couldn’t care less. I had to keep a good condition to work.”* |
|  | During the response to public health emergencies, being able to regulate the frustration and sense of guilt encountered and maintain self-confidence | *“At the beginning of the epidemic, when making calls for epidemiological investigations, I felt very aggravated and frustrated when I asked questions and the other party became impatient. Later on, I became more and more proficient, and became calm and self-confident in dealing with various situations.”* |
|  | During the response to public health emergencies, being able to regulate the fatigue and stress encountered and patiently complete the emergency tasks | *“In the outbreak phase, there was a surge in workload and high stress, when you had to learn to regulate yourself, not to be overwhelmed by all those negative things, and you still needed to be patient to complete the task”* |
| Self-behavioral regulation | During the response to public health emergencies, being able to adjust the role quickly and undertake the emergency tasks within the responsibility | *“Doing things during an epidemic was different from my usual job. Although I usually have training exercises, I have little on-site experience. Now I am assigned emergency tasks that I can complete proficiently and quickly.”* |
|  | During the response to public health emergencies, being able to adjust the programmed operation according to the on-site situation, and be more flexible in completing the emergency tasks | *“At the beginning of the COVID-19 outbreak, there was a surge in isolation and a need to choose centralized isolation sites for medical observation. We wouldn’t be able to follow the guidelines exactly, that could only be handled according to the situation at that time.”* |
|  | During the response to public health emergencies, exchanging and transmitting information actively concerning the on-site situation | *“I always felt that I was inexperienced, and sometimes when I should report the epidemic information, I didn't take the initiative to report it for fear of saying something wrong, and I only said it when others asked me. Later, when I realized it was wrong, I would go and take the initiative to report it myself.”* |
|  | During the response to public health emergencies, being able to take the initiative to put forward epidemic prevention and control opinions according to changes in the situation | *“It needed to carry out a risk assessment and prejudgment, I thought it would be better to manage those quarantine areas separately to reduce the risk of spreading, and I immediately talked to the leader about it.”* |
|  | During the response to public health emergencies, monitoring and analyzing the development trend of public health emergencies proactively | *“In this epidemic prevention and control work, I also no longer wait for others to tell me the situation, I would also take the initiative to observe and learn to analyze on my own.”* |
|  | During the response to public health emergencies, being able to take good personal protection to prevent the spread of dangers | *“To protect ourselves, we unconsciously pressed our masks tighter. There was blood on the top of the nose, to protect yourself and also avoid environmental pollution.”* |
|  | During the response to public health emergencies, being able to proactively provide psychological counseling and health education for the public | *“Later, I slowly learned that I could use some smiles, gestures, and other body language to draw closer to the affected people, so that the work could be carried out more smoothly.”* |
|  | During the response to public health emergencies, being able to direct and cooperate with other personnel in the completion of emergency efforts | *“We didn’t know each other well at that time as a team. Whether you knew each other before or not, since you worked together in the epidemic prevention and control work, you have to cooperate and collaborate.”* |

**Table S3 Main comments and representative quotes from the recovery resilience**

| Sub-theme | Main Comments | Representative quotes |
| --- | --- | --- |
| Adaptive outcomes | After the public health emergency response was completed, being able to quickly return to normal working status | *“After a short period of discomfort, I was able to adjust immediately and get back to the normal work.”* |
|  | After the public health emergency response was completed, summarizing the lessons from the emergencies | *“I once wrote a report on my on-site experience and presented it to the whole organization.”* |
|  | After the public health emergency response was completed, improving and adjusting the ways of routine work according to the lessons from the emergencies | *“I’ve gained a lot of experience, and I still have a lot of work to do better in the future.”* |
|  | After the public health emergency response was completed, conducting evaluative or applied research about the emergencies | *“I have some goals for my research at this stage, and I hope that I can turn my practical experience into theoretical results.”* |

**Table S4 Measurement items to be deleted or revised in the first round of expert consultation**

| No. | Measurement items to be  deleted or revised | The mean value of importance | Coefficient of variation | Revision opinion |
| --- | --- | --- | --- | --- |
| 1 | Ensuring that tasks and resources are coordinated in the health emergency work | 2.958 | 0.323 | It was recommended to delete this item, as the majority of emergency responders’ tasks and resources are carried out according to regulations or assigned by their leaders, and individual coordination plays a minor role in it. |
| 2 | Participating actively in health emergency exercises and training | 3.333 | 0.315 | It was recommended to delete this item as it is the job responsibility of every emergency responder to participate in emergency exercises and training, and does not adequately reflect the resilience connotation. |
| 3 | Learning and training from peers on problems in health emergency work | 3.375 | 0.300 | This item was duplicated by an existing item, and it was suggested that it be revised to “Learning to develop personal emergency communication and interpersonal skills”. |
| 4 | Being able to make use of one’s interpersonal network to obtain professional assistance when needed for health emergency work | 3.833 | 0.295 | It was suggested that it be merged with the next item. |
| 5 | Being able to obtain technical support and operational guidance when encountering difficulties in health emergency work through active personal communication and endeavor | 3.792 | 0.311 | It was suggested that it be merged with the previous item. |
| 6 | Being able to report and analyze events information as required when public health emergencies have just occurred | 3.250 | 0.290 | It was suggested to delete it, as it was a behavioral manifestation and not a stress response. |
| 7 | Being able to quickly and accurately carry out health emergency tasks when public health emergencies have just occurred | 3.292 | 0.341 | It was suggested to delete it, as it was a behavioral manifestation and not a stress response. |
| 8 | During the response to public health emergencies, being more confident in completing the emergency tasks assigned by superiors | 3.208 | 0.318 | It was recommended for deletion, as the cognitive regulation process was not adequately captured. |
| 9 | During the response to public health emergencies, seeing difficult tasks as the accumulation of experience, and making every effort to complete emergency tasks | 3.917 | 0.291 | It was suggested that it be merged with the next item. |
| 10 | During the response to public health emergencies, seeing the stress as an investment in value | 3.875 | 0.326 | It was suggested that it be merged with the previous item. |
| 11 | During the response to public health emergencies, monitoring and analyzing the development trend of public health emergencies proactively | 3.458 | 0.256 | It was suggested to delete this item because professional monitoring and analyses are done by specialized people and are not undertaken by everyone. |
| 12 | During the response to public health emergencies, being able to take good personal protection to prevent the spread of dangers | 3.083 | 0.252 | It was suggested that this item be deleted, as these tasks are also required in the routine work and do not adequately reflect the behavioral adjustments made by emergency responders during public health emergencies. |
| 13 | During the response to public health emergencies, being able to proactively provide psychological counseling and health education for the public | 3.167 | 0.290 | It was suggested that this item be deleted, as these tasks are also required in the routine work and do not adequately reflect the behavioral adjustments made by emergency responders during public health emergencies. |
